# The Impact of Microvascular Decompression on Pain Management in Trigeminal Neuralgia: Clinical Insights

**DOI:** 10.7759/cureus.75987

**Published:** 2024-12-18

**Authors:** Injam Ibrahim Sulaiman, Ahmed Abduljabbar Omar, Sherwan Hussein Hameed

**Affiliations:** 1 Department of Surgery, Hawler Medical University, College of Medicine, Erbil, IRQ; 2 Department of Surgery, Hawler Teaching Hospital, Erbil, IRQ

**Keywords:** effectiveness, erbil, microvascular decompression, pain severity score, trigeminal neuralgia

## Abstract

Background and aim

Trigeminal neuralgia (TN) is a chronic disorder accompanied by recurrent severe pain affecting the face. Many medical regimens are used in the treatment of trigeminal neuralgia. Neurosurgery intervention is the best treatment option, especially with the failure of medical therapy. The aim of the study was to assess the efficacy and outcomes of microvascular decompression for the management of trigeminal neuralgia in Erbil hospitals.

Methods

This is a prospective clinical study done to treat patients with classical or idiopathic (only patients with neurovascular compression) trigeminal neuralgia by microvascular decompression surgery. It was carried out in Hawler Hospital for cardiac and special surgery and Par Hospital in Erbil city, Kurdistan region, Iraq, from April 2023 to February 2024 on a sample of 21 patients who were diagnosed with trigeminal neuralgia.

Results

The preoperative mean pain severity score of patients with TN was 8.9±1.2. The means of pain severity score at early postoperative, two weeks postoperative, two months postoperative, six months postoperative, and twelve months postoperative were 2.5, 1.8, 1.3, 0.7, and 0.4, respectively. The preoperative pain severity score of patients with TN was significantly higher and showed a significant decrease postoperatively. The mean pain severity score of patients with TN was significantly reduced twelve months postoperatively. Postoperative complications were present in 14 (66.7%) patients with TN, commonly paresthesia in nine (42.9%), and hearing symptoms in five (23.8%) patients.

Conclusion

The microvascular decompression is an effective and safe surgical treatment of trigeminal neuralgia.

## Introduction

The trigeminal nerve is the largest cranial nerve, responsible for facial sensation and motor functions such as biting and chewing. This mixed nerve, containing both motor and sensory fibers, originates from the pons and supplies structures of the head and face [1]. Trigeminal neuralgia (TN) was first described in detail in 1756 by Nicholas André, who coined the term "tic douloureux" for the intense pain and facial spasms observed. In 1773, John Fothergill characterized TN as sudden, episodic facial pain triggered by activities like eating or touch, theorizing it might signal a unique disease. In 1891, Sir Victor Horsley performed the first surgical procedure for TN by cutting nerve rootlets. Walter Dandy later identified vascular compression as a cause in 1932, and Peter Jannetta confirmed this in 1967, introducing microvascular decompression, a key treatment still used today [2]. TN causes sudden, electric shock-like facial pain in one or more divisions of the trigeminal nerve. Common triggers include eating, speaking, or light touch. TN predominantly affects women, increases with age, and often involves the right side, possibly due to anatomical factors. Pain is confined to the trigeminal nerve's distribution, excluding areas like the posterior scalp or outer ear. Triggers are often in small sensory zones, and pain may radiate to areas distant from the stimulus site, such as the temple or forehead [3].

The most common etiology of TN is vascular compression at the root entry zone, with the superior cerebellar artery implicated in 85% of cases. Systemic diseases like multiple sclerosis can cause TN primarily through demyelination, while vascular compression is typically caused by conditions such as aneurysms, tumors, or vessel anomalies. Conditions such as hypertension, atherosclerosis, and diabetes may also contribute. Idiopathic cases often follow surgical or dental procedures, with additional factors including structural anomalies like narrow osseous canals and petrous bone irregularities. MRI is essential for identifying underlying causes [4,5].

TN diagnosis is based on patient history, clinical examination, and imaging. Imaging, particularly MRI, plays a crucial role in ruling out other underlying pathologies, such as tumors, arteriovenous malformations, or demyelinating lesions. While TN cannot be diagnosed solely based on imaging, it is essential to identify secondary causes and guide appropriate management. History includes pain timing, location, triggers, severity, and associated conditions. Red flags, such as sensory deficits or bilateral symptoms, warrant further investigation. TN is confirmed by at least three episodes of severe paroxysmal facial pain confined to the trigeminal nerve distribution [3].

The treatment of TN depends on the underlying cause, clinical presentation, and patient factors. Carbamazepine and oxcarbazepine are the first-line treatments, stabilizing neuronal membranes by inhibiting sodium channels. Patients unresponsive to these drugs may receive lamotrigine or baclofen, which targets excitatory neurotransmission, or other antiepileptics like gabapentin, pregabalin, and valproate. For refractory cases, surgical interventions are considered. These include microvascular decompression (MVD), percutaneous rhizotomy, or stereotactic radiosurgery (e.g., Gamma Knife®). The choice of treatment is guided by individual response and the availability of resources [3].

In this study, we aim to assess the efficacy and outcomes of microvascular decompression for the management of trigeminal neuralgia in Erbil hospitals.

## Materials and methods

Study design and settings

A prospective clinical study was done for treating patients with classical or idiopathic (only patients with a neurovascular compression) trigeminal neuralgia by microvascular decompression surgery. The study was carried out in Hawler Hospital for cardiac and special surgery and Par Hospital in Erbil city, Kurdistan region, Iraq, from April of 2023 to Feb of 2024. A total of 21 patients who were diagnosed with trigeminal neuralgia have been admitted to the mentioned hospital for microvascular decompression.

Inclusion and exclusion criteria

The inclusion criteria for the study are as follows: patients diagnosed with classical or idiopathic TN associated with neurovascular compression; clear evidence of vascular compression on 1.5 Tesla MRI following a predefined protocol; preoperative evaluation and follow-up for at least nine months by neurosurgeons or neurologists; cases unresponsive to pharmacotherapy or intolerant to medications; and patients who opted for surgery as the primary treatment. The exclusion criteria include patients with facial pain unrelated to TN, surgeries performed within less than four months, responses to other interventions or medications, and pain scores below 5 or 6.

Sampling

A sample of 21 patients with classical or idiopathic (only patients with a neurovascular compression) trigeminal neuralgia admitted to Hawler Hospital and Par Hospital were selected according to inclusion and exclusion criteria; all patients were operated by microvascular decompression. The data was collected directly by the researcher from patients or from their saved records, and a prepared questionnaire was filled out. 

The questionnaire (see Appendix) was utilized to gather data on several aspects of TN patients. Sociodemographic information included age, gender, and occupation. Clinical characteristics covered disease duration, initial treatment details, subsequent treatments, and pain severity at the first visit. Pain location was also recorded. Preoperative characteristics included pain severity scores, prior interventions, MRI findings, and the duration since MVD. Postoperative outcomes were assessed through complications and pain severity scores at early postoperative stages and at two weeks, two months, six months, and twelve months after surgery. Follow-up details included information on postoperative treatments and their duration.

Neurosurgical evaluation before MVD

The diagnosis of trigeminal neuralgia was implemented by the responsible senior neurologist and based on a detailed history, physical examination, and MRI of the brain with T1-weighted, T2-weighted, and high-resolution 3D constructive interference in steady-state (CISS) or fast imaging employing steady-state acquisition (FIESTA) sequences to detect vascular compression on the trigeminal nerve at its root entry zone. Each patient asked in detail about previous and current treatments with response to medication, dosages and side effects, and previous neurosurgical intervention. For each patient, a visual analog scale assessment was used to determine the severity of pain preoperatively and postoperatively, from 0, meaning no pain, to 10, meaning severe pain.

The preoperative, perioperative, and early postoperative outcomes were measured by means of the questionnaire. Preoperative measurements included data on preoperative conditions and baseline pain scores evaluated at the time of surgical admission. The perioperative measures specifically referred to records made immediately before and after surgery, including pain severity and other vital signs at the time of surgery. Intraoperative data were not reported in this study. Postoperative outcomes had follow-up at two weeks, two months, six months, and up to one year.

Neuroimaging before MVD

All patients underwent MRI examination with sequences and protocols for the detection of vascular compression on the trigeminal nerve, including T1-weighted images to delineate anatomy, T2-weighted images to assess nerve structures and cerebrospinal fluid (CSF), and high-resolution 3D constructive interference in steady-state or FIESTA sequences for clear delineation of the trigeminal nerve and its relationship to adjacent vessels. The MRI available in our area is 1.5 Tesla by Siemens and Philips. Vascular compression was assessed for morphological changes, including contact, displacement, distortion, or atrophy of the trigeminal nerve.

Postoperative follow-up

The patients had to stay at the hospital for at least three days and then were accordingly discharged to home with proper medications. Patients were prospectively assessed by a neurosurgeon at two weeks and nine months after MVD, either at clinic visits or by telephone interviews. At each visit, the scale of pain was recorded.

Each patient was monitored and queried in detail at the predefined follow-up intervals. Major complications included severe outcomes such as death, cerebellar or brainstem infarction, cerebellar or brainstem hemorrhage, anesthesia dolorosa, meningitis, cerebrospinal fluid leak, hydrocephalus, permanent ataxia, diplopia, corneal keratitis, severe hypoesthesia, facial weakness or nerve palsy, and permanent hearing loss or impairment. Minor complications encompassed dizziness, tinnitus, fatigue, wound infection, headache, scar tissue pain, transient ataxia, diplopia, mild or severe hypoesthesia (transient or permanent), trigeminal motor weakness, transient facial weakness, transient hearing loss or impairment, and altered taste sensation.

Ethical considerations

Ethical approval for the study was obtained from Hawler Medical University board number 14 on the 9^th^ of March 2023, and official agreements were secured from hospital authorities. Verbal and written consent was obtained from patients after explaining the study's aim and ensuring confidentiality.

Statistical analysis 

Statistical Package for Social Sciences (SPSS) version 22 was used for data entry and analysis. Continuous variables were presented as mean ± standard deviation (SD), and categorical variables were presented as frequencies and percentages. Multiple contingency tables were conducted, and appropriate statistical tests were performed; a paired t-test was used to compare two consecutive means, while repeated measures analysis was used in the comparison between more than two consecutive means. In all statistical analyses, the level of significance (p-value) was set at ≤0.05.

## Results

This study included 21 patients with TN. The mean age was 51.2±11.2 years, ranging from 33 to 69 years. Female patients were more common than males (57.1% vs. 42.9%). The predominant occupation among patients was a housewife (38.1%), as shown in Table 1.

**Table 1:** Sociodemographic characteristics of patients with trigeminal neuralgia (TN)

Variable	n	%
Age		
<40 years	3	14.3
40-49 years	6	28.6
50-59 years	6	28.6
≥60 years	6	28.6
Gender		
Male	9	42.9
Female	12	57.1
Occupation		
Housewife	8	38.1
Public servant	5	23.8
Self-employed	5	23.8
Retired	3	14.3
Total	21	100.0

The mean duration of TN before surgery was six years. The majority of patients (n=14, 66.7%) had a disease duration of five years or more. Carbamazepine was the most common first-line treatment. Further details on treatment and disease duration are provided in Table 2.

**Table 2:** Clinical characteristics of patients with trigeminal neuralgia (TN)

Variable	n	%
Disease duration (mean±SD): 6±3 years		
<5 years	7	33.3
≥5 years	14	66.7
First treatment		
Carbamazepine 200 mg	15	71.4
Carbamazepine 400 mg	5	23.8
Pregabalin 75 mg	1	4.8
Duration of first treatment (mean±SD): 16±14.9 months		
<1 year	10	47.6
≥1 year	11	52.4
Second treatment		
No	4	19.0
Gabapentin	9	42.9
Pregabalin	5	23.8
Phenytoin sodium	1	4.8
Gabapentin and phenytoin sodium	1	4.8
Gabapentin and amitriptyline	1	4.8
First visit pain severity score (mean±SD): 8±1.4		
Total	21	100.0

The most common pain location involved two branches (V2 and V3), as detailed in Table 3.

**Table 3:** Pain location of patients with trigeminal neuralgia (TN)

Variable	n	%
Pain location		
Right-sided two branches pain (V2 and V3)	5	23.8
Left-sided two branches pain (V2 and V3)	9	42.9
Bilateral two branches pain (V2 and V3)	1	4.8
Right-sided V2 branch	1	4.8
Left-sided V2 branch	3	14.1
Right-sided V3 branch	1	4.8
Left-sided three branches (V1, V2 and V3)	1	4.8
Total	21	100.0

The mean preoperative pain severity score of the patients in the TN group was 8.9. Vascular compression on MRI findings confirmed the diagnosis of neurovascular conflict in 85.8% (n=18) of the patients. Preinterventions mentioned by some patients included injections, radiofrequency ablation, and Gamma Knife® procedures. However, there was no prior intervention in seven cases (33.3%). The time duration since microvascular decompression surgery was quite varying; the details can be seen in Table 4.

**Table 4:** Preoperative characteristics of patients with trigeminal neuralgia (TN) MVD - microvascular decompression

Variable	n	%
Preoperative pain severity score (mean±SD): 8.9±1.2		
Any other intervention		
No	7	33.3
Injection	6	28.6
Radiofrequency	1	4.8
Gamma Knife®	3	14.2
Injection, radiofrequency and Gamma Knife®	1	4.8
Injection and radiofrequency	2	9.5
Injection and Gamma Knife®	1	4.8
MRI findings		
Normal	3	14.2
Vascular compression	18	85.8
Duration since MVD operation (mean±SD): 11.9±8.9 months		
<1 year	11	52.4
≥1 year	10	47.6
Total	21	100.0

Postoperative complications were present in 14 (66.7%) patients with TN; commonly paresthesia in six (42.9%) patients, hearing symptoms in three (21.6%) patients, followed by one patient (7.10%) with CSF leak, paresthesia and psychosis in one (7.1%) patient, CSF leak, paresthesia and skin infection in one (7.1%) patient, paresthesia and facial symptoms in one (7.1%) patient and paresthesia and double vision also in one (7.1%) patient. The means of pain severity score at early postoperative, two weeks postoperative, two months postoperative, six months postoperative, and twelve months postoperative were 2.5, 1.8, 1.3, 0.7, and 0.4, respectively (see Table 5).

**Table 5:** Postoperative outcomes of patients with trigeminal neuralgia (TN) CSF - cerebrospinal fluid

Variable	n	%
Postoperative complications		
Yes	14	66.7
No	7	33.3
Complication types		
CSF leak	1	7.1
Paresthesia	6	42.9
Hearing symptoms (vertigo, dizziness)	3	21.6
Paresthesia and psychosis	1	7.1
CSF leak, paresthesia and skin infection	1	7.1
Paresthesia and facial symptoms	1	7.1
Paresthesia and double vision	1	7.1
Twelve months postoperative pain severity score (mean±SD): 0.4±1.1		

The common follow-up treatment was carbamazepine 200 mg for 12 (57.1%) patients, followed by carbamazepine 100 mg for three (14.2%) patients, carbamazepine 200 mg and phenytoin sodium 50 mg for two (9.5%) patients, carbamazepine 100 mg and gabapentin 300 mg for one (4.8%) patient, carbamazepine 100 mg then phenytoin 50 mg for one (4.8%), carbamazepine 400 mg, then gabapentin cap 300 mg for one (4.8%) and carbamazepine 200 mg and amitriptyline 25 mg also for one (4.8%) patient. The mean duration of postoperative treatment was 3.4 months; 18 (85.7%) of patients had a mean follow-up duration of six months or less (see Table 6).

**Table 6:** Follow up characteristics of patients with trigeminal neuralgia (TN)

Variable	n	%
Follow-up treatment		
Carbamazepine 100 mg	3	14.3
Carbamazepine 200 mg	12	57.1
Carbamazepine 100 mg and gabapentin 300 mg	1	4.8
Carbamazepine 100 mg and phenytoin sodium 50 mg	1	4.8
Carbamazepine 400 mg, then gabapentin300 mg without response	1	4.8
Carbamazepine 200 mg and phenytoin 50 mg	2	9.5
Carbamazepine 200 mg and amitriptyline 25 mg	1	4.8
Duration of postoperative treatment (mean±SD): 3.4±3.1 months		
≤6 months	18	85.7
>6 months	3	14.3
Total	21	100.0

The pain severity score of patients with TN was significantly increased at preoperative measurement compared to the score recorded at the first visit (p=0.003), as detailed in Table 7. 

**Table 7:** Distribution of pain severity score of trigeminal neuralgia (TN) at first visit and preoperative measurement Paired t-test, significant at the p-value of ≤0.05.

Variable	Study period		p-value
	First visit (mean±SD)	Preoperative (mean±SD)	
Pain score	8±1.4	8.9±1.2	0.003

## Discussion

TN remains one of the most debilitating craniofacial pain disorders, often necessitating surgical intervention when medical therapy fails. A study conducted in Iraq highlighted the efficacy of MVD as a gold-standard surgical approach, reporting over 95% pain relief within six months of follow-up. This procedure demonstrated significant reductions in pain scores, particularly in cases involving vascular compression at the trigeminal nerve's root entry zone, reaffirming its role as a cornerstone treatment for refractory TN​ [6].

In the current study, the mean age of patients with TN was 51.4 years. This mean age is close to the mean age of 50.7 years, as reported by Ayele et al. (2020) cross-sectional study in Ethiopia [7]. Our study showed that female patients with TN were more than males 12 vs 9 (57.1% vs. 42.9%). This finding coincides with De Stefano et al. (2023) cross-sectional study in Italy, which revealed that the female gender was prevalent in TN, while the male gender was predictive of TN clinical co-morbidities [8]. 

In our series, 71.4% of the patients were started on carbamazepine 200 mg and titrated up depending on tolerance and efficacy. However, before surgery, the total dose for most patients reached an average maximum of 400-800 mg. Several patients, despite dose escalation, developed recurrence of pain or showed significant side effects in the form of dizziness and drowsiness, for which they had to discontinue their treatment. Besides, some patients could not continue medical management because of financial constraints. These factors necessitated surgical intervention. These findings correspond with the results of Mohsin et al. (2019) study done in Iraq, which reported that carbamazepine 200 mg tablet was the first-line of medical treatment for patients with trigeminal neuralgia [9]. In our study, the mean duration of the first treatment was 16 months; eight (52.4%) patients had one year and more for the first treatment duration. This finding is close to the results reported in Obermann's study (2020) in Germany [10]. Our study found that the second treatment was administered for 17 (81%) patients, commonly gabapentin, pregabalin, etc. These findings are similar to a study by Xu et al. (2021) in the United States of America, which reported that gabapentin and pregabalin are the common second-line treatment of trigeminal neuralgia [11].

The present study showed that the common pain location of trigeminal neuralgia was left-sided two-branch pain (V2 and V3). In our study, other interventions were absent in seven (33.3%) patients with TN, while other interventions were mainly injection in six (28.6%) patients, Gamma Knife® in three (14.2%) patients, injection and radiofrequency in two (9.5%) patients, etc. The patients who had undergone Gamma Knife® treatment received it at least one year prior to surgery. These findings are in agreement with the results of the study by Koopman et al. (2011) in the Netherlands [12]. Our study found that MRI showed vascular compression in 18 (85.8%) patients with TN. Among the 21 patients included in the study, three had undergone prior Gamma Knife® radiosurgery. These patients were selected because they met the inclusion criteria of persistent or recurrent pain despite previous interventions, making them eligible for microvascular decompression surgery. This finding is parallel to the results of the study by Hughes et al. (2016) conducted in the United States of America [13]. The current study showed that the pain severity score of patients with TN was significantly increased at preoperative measurement from the first visit (p=0.003). Consistently, Danyluk et al. (2021) prospective study in Canada revealed that failure of medical treatment led to an increase in pain score during the preoperative period compared to the first visit pain score [14].

This study showed that the mean pain severity score of patients with TN postoperatively was significantly reduced after twelve months (p<0.001). This finding is similar to many other results, such as the study by Paulo et al. (2020) in the United States of America, which found a significant reduction in pain severity score for TN after 12 months following MVD [15]. Another study conducted in China by Zhao et al. (2018) found that MVD was a safe and effective surgical option in the management of trigeminal neuralgia [16].

In the present study, postoperative complications were present in 14 (66.7%) patients with TN, commonly paraesthesia and hearing symptoms. These findings are similar to the results of Andersen et al. (2022) prospective study in Denmark, which found that postoperative complications of MVD were present in about two-thirds of patients with TN, commonly paraesthesia and hearing symptoms [17]. A randomized prospective study conducted in Iraq by Arkawazi and Faraj found that the MVD stills as the standard surgical method of treatment for patients with TN with a better pain relief rate, lesser pain recurrence rate, and faster response than Gamma Knife® radiosurgery [18]. In our study, a common follow-up treatment was carbamazepine (Tegretol®) 200 mg.

Study limitations

Several limitations of this study must be acknowledged. Firstly, the sample size of only 21 patients is relatively small and may limit the generalizability of these findings. Secondly, the investigation was carried out in the region of Erbil, Iraq; thus, the application of these results to other populations could be limited. Thirdly, long-term follow-up beyond 12 months may not capture delayed complications or pain recurrence. This also introduces some subjectivity to the assessment of outcomes due to the use of self-reported pain scores. Lastly, these results will have to be further verified in future multi-center studies using larger samples with extended follow-up.

## Conclusions

Microvascular decompression is a safe and effective surgical treatment for trigeminal neuralgia, significantly reducing pain scores 12 months postoperatively. Common postoperative complications include paresthesia and hearing-related symptoms. Carbamazepine remains an effective follow-up treatment after surgery. Neurosurgeons are encouraged to consider microvascular decompression as the first-line surgical option for trigeminal neuralgia. Postoperative complications should be carefully monitored, and further national multi-center studies are recommended to evaluate long-term outcomes.

## Appendices

**Table 8:** Comprehensive questionnaire for patients assessment CSF - cerebrospinal fluid; MVD - microvascular decompression

Question	Response
Name	...........................................................................
Age	...........................................................................
Sex	...........................................................................
Occupation	...........................................................................
Date of 1st diagnosis (symptoms)	...........................................................................
First treatment with good pain relief	...........................................................................
Dose	...........................................................................
Duration	...........................................................................
2nd + 3rd treatment	...........................................................................
Severity of pain on first visit (score 0–10)	...........................................................................
Severity of pain before operation (score 0–10)	...........................................................................
Any other Interventions:	
- Injection	...........................................................................
- Radiofrequency	...........................................................................
- Gamma Knife	...........................................................................
- Others	...........................................................................
Image findings	...........................................................................
Date of MVD operation	...........................................................................
Post-operative complications:	
- CSF leak	...........................................................................
- Paresthesia	...........................................................................
- Hearing symptoms	...........................................................................
- Facial symptoms	...........................................................................
- Other cranial nerve complications	...........................................................................
- Meningitis	...........................................................................
- Skin infection	...........................................................................
- Other complications	...........................................................................
Post-operative pain score (0–10, 0–3 days)	...........................................................................
Follow-up pain score (0–10):	
- 2 weeks	...........................................................................
- 2 months	...........................................................................
- 6 months	...........................................................................
- 12 months	...........................................................................
- 18 months	...........................................................................
- 2 years	...........................................................................
- 3 years	...........................................................................
